# Editorial

**Published:** 1867-01

**Authors:** 


### Editorial.

### CHICAGO MEDICAL SOCIETY.

JAN. 18th. The regular meeting of the Society was almost wholly occupied with the subject of City Health Regulations. Dr. N. S. DAVIS, from the Special Committee on that subject, made the following report, which, after free discussion, was adopted. The Committee was instructed to present the same to the Common Council, with the draft of an amendment to the present laws, as indicated in the report.

### REPORT OF THE COMMITTEE ON THE HEALTH DEPARTMENT OF OUR CITY GOVERNMENT.

Presented to the Chicago Medical Society, January 18th, 1867.

The subject assigned to your Committee for consideration, is certainly one of the most important that can engage the attention of the people of this, or any other large city. The adoption and enforcement of proper sanitary regulations is so closely connected with the preservation of health and the prolongation of life and happiness, that no community can neglect them without, sooner or later, suffering the severest penalties. In fulfilling the duties assigned to your Committee, three questions require careful consideration.

1st. What are principles upon which a Municipal Health Department should be organized, in order to ensure the highest degree of enlightment and efficient action?

2d. Is the present organization of the Health Department of our city defective; and if so, in what particulars?

3d. What changes are necessary to remedy its defects, and complete its efficiency?

In answer to the first question, we would state, that the basis of a proper Health Organization, should consist of a Board of Commissioners, embodying in its members a high degree of executive ability or business capacity, and a thorough knowledge of *sanitary science*, as applied to the preservation of the health of communities and the prevention of the spread of contagious diseases. No board can embody these qualities, without being composed in part, at least, of thoroughly-educated physicians; simply because, in our country, no other class of men are educated to an adequate extent, in reference to those sanitary subjects on which a Board of Health must continually act. The truth of this proposition is too plain to need either argument or illustration.

A Board of Health Commissioners, composed of properly educated and qualified members, should be invested with sufficient power to devise and carry into prompt effect, all such measures as the safety and welfare of the community requires. The term of office of its members should be long enough to insure a reasonable degree of permanency in whatever sanitary measures or policy may be adopted. In no department of municipal affairs, is a vacillating, temporizing policy more disastrous than in that relating to the preservation of the public health. A show of energy, or a display of zeal, or a prodigal expenditure of money just while some fearful epidemic disease is threatening to commence its work of death, and a negligent indifference at all other times, is but little better than no action at all. Sanitary measures, to be successful in lessening the sickness and mortality of communities, and in protecting them from the ravages of epidemics, must be founded on an accurate knowledge of all those local causes that tend to deteriorate the public health, and then they must be rigidly and perseveringly enforced from year to year.

To enable a Board of Health Commissioners to devise such measures, and establish an enlightened and permanent sanitary policy, its members should not only possess the requisite education and business capacity, with a term of office long enough to add experience to their previous knowledge, but they should be appointed in such a manner as to free them as far as possible from all partisanship, or feelings of dependence on the success or failure of mere political parties. And yet they should be fully accountable to the municipal government for all their official acts. Such are the principles on which a Board of Health Commissioners should be organized.

The next question is, how far the present health department of our city government is organized in accordance with these principles, and in what particulars is it defective? Our present Health Department consists of a Board of three Commissioners, who are also at the same time Commissioners of the Police and Fire Departments; a Health-Officer; and a City Physician. The three members of the Board are elected by the people, one from each division of the city, and hold their offices for six years. The Health-Officer is appointed by the Board. The City Physician is elected by the Common Council, and holds office for two years.

The present city charter confers on the Common Council power "to do all acts and make all regulations which may be necessary or expedient, for the preservation of health, and the suppression of disease." And, in accordance with this power, we find in the present laws and ordinances of the city full and ample powers conferred and duties enjoined upon the Board of of Health and Health-Officer, for the efficient and thorough regulation of everything relating to the public health and safety. After a full examination of the present laws and ordinances, we can see no need of further legislation, so far as relates to the conferring of power upon the Board of Health or its agents.

Wherein, then, is the present organization defective? Chiefly in three important particulars. The first and most radical defect is, the absence of all provision for securing the selection of members of the Board of Health so thoroughly educated in sanitary matters as to fit them for an enlightened and efficient discharge of their duties. To make up a Board of Health, charged with the duty of making and enforcing all such regulations as are necessary to mitigate or prevent the prevalence of diseases in a great city, of men who, neither by education, occupation, nor business habits, have acquired any special knowledge of the nature and causes of disease, or of the laws by which they are developed and diffused, is just as absurd as it would be to elect a practising physician to the office of City Attorney. If there is lack of efficiency in the practical working of our present health organization, it is not because the members of the present Board of Police, acting as a Board of Health, are deficient in integrity or business capacity, or from deficient legal authority to act, but simply because they have not that special sanitary or medical education which enables them to comprehend clearly the nature, extent, and importance of the work entrusted to them.

The second defect consists in an imperfect and injudicious distribution of duties between the Health-Officer, City Physician, and Clerk of the Board of Health. The Health-Officer shall be simply an intelligent and efficient executive officer, to superintend the prompt enforcement of all the laws and orders of the Board of Health. The City Physician should keep at all times a free *vaccine* dispensary for the poor, and have the immediate charge or superintendence of such sick persons as came under the care and authority of the Health and Police Departments of the City Government. And there should be a competent Clerk, who should perform the duties of Secretary to the Board of Health and the Health-Officer, and also the duties of Register of Vital Statistics.

The third defect in the present organization is the absence of efficient regulations for the accurate registry of births, marriages and deaths, with the causes of the latter. The importance of this needs no comment here. After a somewhat careful and candid examination of the whole subject, we feel certain that whatever inefficiency or defectiveness there is in the practical working of the present health organization of our city, can be traced directly or indirectly to the three sources just enumerated. This leads us to the third and last question involved in this report, namely: What changes are necessary to remedy the defects to which we have alluded? We answer, so far as State legislation is concerned, we need but a single brief amendment to the laws now in force. If the present law relating to the action of the Board of Police, in the capacity of a Board of Health, was so amended as to provide for the appointment of three thoroughly competent Physicians, (one from each Division of the City,) to act with said Police Commissioners in all matters pertaining to health or sanitary regulations, to possess coëqual powers and duties, and to constitute a part of said Board whenever acting in the capacity of a Board of Health, it would be all the legislation really required. This done, the other defects pointed out could be easily remedied under the authority already possessed by the Common Council and Board of Health. While the proposed amendment should leave the present mode of electing the three Police Commissioners undisturbed, it should provide some other and less political method of appointing the *three* medical members of the Board acting as a Board of Health. Those members of our profession who have acquired that education, experience, and reputation, which would enable them to impart that kind of intelligence and efficiency to a Board of Health, imperatively needed in all large cities, will never be found seeking nominations from political parties, nor lobbying for appointments by a Governor and Senate, or a Mayor and Council. They must be sought ought and invited to accept the position, or their services will not be obtained. We would suggest whether this would not be done more judiciously and with less reference to anything of a political or partisan character, by the Judges of the Superior Court of this city, than by any other authority. It will be seen that the defects we have pointed out and the remedies proposed are few and simple; yet they are really of vital importance to the practical working and beneficial results of the Health Department of our city.

Should the views thus far expressed in this report meet the approbation of this Society, an amendment could be prepared in due form, relating to the appointment of three competent medical men as members of the Board of Health, and an ordinance in relation to the proper registry of vital statistics, and we have no doubt but both would meet the prompt sanction of the Mayor and Council. Before concluding this report, it may be expected that some notice will be taken of the several amendments and health bills which have already been prepared and placed before the Legislature, the Council, and the public. There are three separate projects of this kind. The first consists in amendments to the present laws prepared and recommended by the Common Council. The only item in these, that relates to the important defects we have explained, is the section proposing to give the Common Council power, in cases of imminent danger from the prevalence of epidemic diseases, to appoint an additional number of members of the Board of Health, to act as such simply while such danger lasts. But it is not provided that even these temporary appointees shall be medical men. And hence it does not in any degree remedy the radical defect in the constitution of of the present Board. The second project is in the form of a bill recently introduced into the State Senate by Senator Ward. By the provisions of this bill, everything pertaining to the Health Department, and sanitary regulations, is removed from the control of the people of the city and its municipal government, and placed in the hands of a Board of Commissioners, consisting of the President of the Board of Police, and two Commissioners appointed by the Governor and Senate, one of whom must be a physician. The two persons thus appointed by the Governor and Senate are to hold office four years, and, with the President of the Board of Police, constitute a Board of Health, with full power to make all needful rules, regulations, and appointments of agents, etc., sufficient to devise and execute a complete and independent sanitary system. There are two leading and fatal objections to this bill. The first is, that it deprives the people of this city of all power to regulate and control some of their most important local interests, and assumes that a Governor and Senate, at Springfield, are better qualified to judge of the capacities and qualifications for office, of citizens of Chicago, than are the local authorities of the city, or the people themselves. The second, and more important objection is, that it creates a large number of new and additional officers and employés, requiring a regular annual additional tax upon the city, of from $75,000 to $150,000, without regard to any *extraordinary* expenditures on account of the prevalence of severe epidemics. And yet it does not provide for the accomplishment of a single valuable object, that would not be as well and certainly accomplished, by simply adding *hree* thoroughly competent physicians to our present Board of Health. Serious objections could also be made to several items in the details of this bill. For instance, the section relating to the registration of births, marriages, and deaths, is utterly worthless.

The third project is that presented by the Citizens' Committee, and entitled the "Metropolitan Health Bill." This is amenable to precisely the same objections as we have made to the bill introduced by Senator Ward; while, practically, it would be far less efficient. Like Ward's bill, it takes from the people and local authorities the selection of a Board of Health, and confers it on the Governor and Senate, at Springfield. Like Ward's bill, it creates a long list of new and additional city officers and employees, requiring a correspondingly large additional expenditure of money annually. But, unlike Ward's bill, it does not fix the salary of the members of the Board of Health, or provide for the appointment of a full corps of Sanitary Police. It allows the Common Council to fix their salaries, thereby making the officers perform the difficult task of serving two masters; the Governor for their appointment, and the Council for their pay. Again, by depending in part upon the ordinary police for executing the orders of the Health Board, it places the police in the equally embarassing position of obedience to the orders of two separate and independent Boards—a plan that never has and never will work satisfactorily in practice.

In conclusion, we cannot refrain from again expressing the opinion, that a single amendment to our present laws, providing for the addition of *three* competent and thoroughly educated members of the medical profession to the present Board of Health, with the aid of one additional Clerk, as Register of Vital Statistics, would result in rendering our Health Department as efficient and beneficial to the interests of the city, as it could be by any number of new and expensive schemes. It would require but one police organization, capable of being increased or diminished, as public exigencies might require, and practically amenable to but one Board.

It would require a larger ratio of medical to the non-medical intelligence in the Board of Health, than is proposed in either WARD'S bill, or that of the Citizens' Committee. Instead of requiring an annual aggregate increased tax upon the city of $75,000 or $100,000 for salaries of new officers and agents, it would require for the *three* additional members of the Board of Health and the Register of Vital Statistics an aggregate of not over $5,000. It would also leave our City Government far less complicated in its details, and, in all its strictly local interests, under the control of its own citizens, where it properly belongs.

All of which is respectfully submitted.

N. S. DAVIS,<br>R. C. HAMILL,<br>J. P. ROSS, } Committee.

---

MEDICAL DEPARTMENT OF UNIVERSITY OF MICHIGAN.—We have seen in some of our exchanges, a statement of the number of Medical Students in the University of Michigan, accompanied by the assertion that the large number congregated there could not have been drawn thither by the small pecuniary charges, as the aggregate of attendance is greater, owing to the length of the Lecture-term, than in any other school in the North-west. The entire falsity of such a statement is shown by the following facts:—

The fee for admission to the Medical Department of the Michigan University is, for students in the State $10, from out of the State $20, paid but once. The Lecture-term being six months, allowing $25 per month for board, would make a total necessary expenditure for the full term of $160 for the student of the State, and $170 for students from other States. The Chicago Medical College, in this city, has a Lecture-term of full five months. If we put board here at the same rate as supposed for Ann Arbor, namely, $25 per month, the cost of attending the regular annual course here, would be as follows:—

| | |
|---|---|
| Five months board, at $25 per month, ---- | $ 125 00 |
| Lecture fees, ---- | 50 00 |
| Matriculation fee, ---- | 5 00 |
| Dissecting fee, ---- | 5 00 |
| Hospital fee, ---- | 6 00 |
| Total, ---- | $191 00 |

---

Clinical Items.—A subscriber wishes to know how to cure "an obstinate, tormenting, intolerable itching, of years standing," either in the genital organs of the female or around the anus of the male.

The following cases may answer his purpose:—

Case I. Mrs. B., a married lady, aged about 25 years, had been for several years subject to periodical attacks of puritus pudendi, or intolerable itching of the labia and vulva. She generally suffered most from it after the menstrual periods, and it was generally accompanied by a thin leucorrhœal discharge. She was placed on the following treatment:—

| | | |
|---|---|---|
| ℞. | Ext. Hyoscyamus, ---- | 30grs. |
| | Sulph Ferri, ---- | 30grs. |
| | Pulv. Aloes, ---- | 15grs. |
| | Blue Mass, ---- | 10grs. |
| | Ext. Nux Vom., ---- | 10grs. |

Mix and divide into thirty pills. One to be taken before breakfast and dinner each day. For local use she was directed a solution of borate of soda (borax), ℨiij and sulphate of morphia, 20grs., in water, one pint, the vulva to be wet with it often, and a small quantity injected into the vagina each night and morning. Under this treatment the patient recovered, without any return of the disease.

CASE II. Mrs. W., aged 40 years, had been very severely troubled with the same disease several months, without any regard to the menstrual periods. The same solution of borax and morphine, applied locally, and the use of eight drops of Fowlers's arsenical solution, taken before each meal, in a spoonful of sweetened water, and continued for about four weeks, resulted in a cure. Both patients were required to live on plain food and to avoid all stimulating drinks.

CASE III. Mr. G., aged 30 years, sanguine temperament and full habit, had pruritis of the anus for three years. Sometimes the itching would be intolerable for several days and then better for a week or ten days at a time. On examination, the skin, over a circle of an inch, around the opening of the rectum, was thickened, slightly fissured, and whiter than usual. He was directed to take eight drops of Donovan's solution before each meal time, and the affected surface was wet with the liquid persulphate of iron, of the strength usually found in the drug stores, every third day. After four local applications he was entirely relieved. He continued to take the drops internally for three weeks, and although more than one year has elapsed the disease has not returned.

I recollect treating several cases of old chronic cases of pruritis ani sucessfully, by applying locally, each night and morning, the following ointment:

℞. Iodide Sulphur,------------------------- ℈ij
Oil Tobacco, ------------------------------ 2gtts.
Simple Crete,------------------------------ ℥ij

Attention should always be paid to the general health, and especially to the condition of the digestive organs.

---

THE CHAIR OF SURGERY IN RUSH MEDICAL COLLEGE.—According to statements in the daily papers of this city, the chair of Surgery made vacant by the death of the late Prof. D. BRAINARD, has been filled by the appointment of MOSES GUNN, of Detroit, Professor of Surgery in the University of Michigan. It is also stated that Prof. GUNN has accepted the appointment.

---

CITY MORTALITY.—The following is the report of the Health-Officer of the mortality of the City of Chicago for the month of December, 1866:

CAUSES OF DEATH.

| | | | |
|---|---|---|---|
| Accidents, | 4 | Fever, Scarlet, | 13 |
| Asthma, | 2 | Fever, Typhoid, | 4 |
| Bronchitis, | 1 | Fever, Typhus, | 1 |
| Burned, | 1 | Fever, not stated, | 1 |
| Cancer, | 2 | Hydrocephelus, | 7 |
| Childbed, | 2 | Inflammation of Kidneys, | 1 |
| Cholera Infantum, | 1 | Inflammation of Bowels, | 9 |
| Consumption, | 42 | Inflammation of Lungs, | 6 |
| Convulsions, | 35 | Inflammation of Liver, | 1 |
| Croup, | 7 | Marasmus, | 1 |
| Cold, | 2 | Old Age, | 15 |
| Chicken Pox, | 1 | Palsy, | 2 |
| Congestion of Brain, | 3 | Pneumonia, | 7 |
| Congestion of Lungs, | 2 | Phthisis Pulmonalis, | 1 |
| Decline, | 1 | Spasms, | 1 |
| Delirium Tremens, | 1 | Spinal Meningitis, | 1 |
| Diarrhœa, | 5 | Suffocation, | 2 |
| Diphtheria, | 15 | Small Pox, | 2 |
| Disease of Heart, | 8 | Suicide, | 1 |
| Disease of Liver, | 2 | Stillborn, | 10 |
| Disease of Lungs, | 7 | Teething, | 3 |
| Disease of Brain, | 6 | Whooping-Cough, | 17 |
| Dropsy, | 7 | Gun shot Wound, | 1 |
| Drowned, | 2 | White Swelling, | 1 |
| Erysipelas, | 3 | Unknown, | 33 |
| Fever, Childbed, | 2 | | |
| Fever, Remittent, | 4 | Total, | 309 |

AGES OF THE DECEASED.—Under 5 years, 153; over 5 and under 10 years, 19; over 10 and under 20, 15; over 20 and under 30, 28; over 30 and under 40, 34; over 40 and under 50, 16; over 50 and under 60, 11; over 60 and under 70, 11; over 70 and under 80, 12; over 80 and under 90, 6; unknown, 4. Total, 309.

NATIVITIES.

| | | | | | |
|---|---|---|---|---|---|
| Chicago, | 128 | England, | 5 | Sweden, | 5 |
| Other States, | 63 | Germany, | 47 | Scotland, | 4 |
| Belgium, | 1 | Ireland, | 38 | Wales, | 1 |
| Bohemia, | 2 | Norway, | 6 | Unknown, | 6 |
| Canada, | 2 | On the Sea, | 1 | Total, | 309 |

DIVISIONS OF THE CITY.

North, ......... 75 | South, ......... 105 | West, .......... 129 | Total, ......... 309

Total number during the month of November, 382

Decrease from last month, 73

Total number last year for the month of December, 333

---

DR. CONNEAU.—It has been remarked that nearly every profession but that of medicine was represented in the French Senate. This anomaly has struck the Emperor, it would appear, as the *Evenement* announces that his Majesty's physician, Dr. Conneau, is to be promoted forthwith to a seat at the Luxembourg.

